# Emulsion Stabilization by Cationic Lignin Surfactants Derived from Bioethanol Production and Kraft Pulping Processes

**DOI:** 10.3390/polym14142879

**Published:** 2022-07-15

**Authors:** Avido Yuliestyan, Pedro Partal, Francisco J. Navarro, Raquel Martín-Sampedro, David Ibarra, María E. Eugenio

**Affiliations:** 1 Department of Chemical Engineering, Faculty of Industrial Technology, UPN Veteran Yogyakarta, Jalan SWK 104, Yogyakarta 55283, Indonesia; avido.yuliestyan@upnyk.ac.id; 2 Pro2TecS-Chemical Process and Product Technology Research Centre, Department of Chemical Engineeing, ETSI, Campus de “El Carmen”, University of Huelva, 21071 Huelva, Spain; frando@uhu.es; 3 Forest Research Center (INIA, CSIC), Ctra. de la Coruña km 7.5, 28040 Madrid, Spain; raquel.martin@inia.csic.es (R.M.-S.); ibarra.david@inia.csic.es (D.I.); mariaeugenia@inia.csic.es (M.E.E.)

**Keywords:** lignin, amination, emulsion, bitumen, rheology, microstructure

## Abstract

Oil-in-water bitumen emulsions stabilized by biobased surfactants such as lignin are in line with the current sustainable approaches of the asphalt industry involving bitumen emulsions for reduced temperature asphalt technologies. With this aim, three lignins, derived from the kraft pulping and bioethanol industries, were chemically modified via the Mannich reaction to be used as cationic emulsifiers. A comprehensive chemical characterization was conducted on raw lignin-rich products, showing that the kraft sample presents a higher lignin concentration and lower molecular weight. Instead, bioethanol-derived samples, with characteristics of non-woody lignins, present a high concentration of carbohydrate residues and ashes. Lignin amination was performed at pH = 10 and 13, using tetraethylene pentamine and formaldehyde as reagents at three different stoichiometric molar ratios. The emulsification ability of such cationic surfactants was firstly studied on prototype silicone oil-in-water emulsions, attending to their droplet size distribution and viscous behavior. Among the synthetized surfactants, cationic kraft lignin has shown the best emulsification performance, being used for the development of bitumen emulsions. In this regard, cationic kraft lignin has successfully stabilized oil-in-water emulsions containing 60% bitumen using small surfactant concentrations, between 0.25 and 0.75%, which was obtained at pH = 13 and reagent molar ratios between 1/7/7 and 1/28/28 (lignin/tetraethylene pentamine/formaldehyde).

## 1. Introduction

Lignin is after cellulose the second most abundant renewable polymer [1,2], being mainly synthetized by enzymatic polymerization of the following monomers: p-coumaryl alcohol, coniferyl alcohol, and synapyl alcohol. Each of these monolignols gives rise to different types of lignin units, which are called p-hydroxyphenyl (H), guaiacyl (G), and syringyl (S), respectively [3]. It is known that other monomers such as coniferaldehyde, acylated monolignols, etc. could also act as precursors of lignin [3]. The final polymeric structure of lignin is a heterogeneous complex macromolecule with different types of bonds and a large variety of functional groups that depend on the biomass source [4]. Accordingly, hardwood would contain approximately 20–25% of lignin, which is mostly composed of G and S units and traces of H units. On the other hand, softwood with around 20–35% of lignin mostly contains G units and small amount of H units [5,6]. Finally, non-woody species have lower lignin content (9–20%) of HGS type, which is characterized by a high proportion of H units [7]. Moreover, along with their source, the method used to generate the different lignins strongly affects their characteristics and, consequently, industrial applications [8,9].

Nowadays, pulp and paper industries, using processes such as kraft and sulfite pulping, generate a high amount of residual lignin dissolved in the black liquor, yielding kraft lignin and lignosulfonates [10]. Around 50–70 million tons/year of lignin are generated by this industry, mainly from kraft mills, since it is the most extended pulping technology to produce cellulose pulp [11,12]. On the other hand, the residues generated in lignocellulosic biorefineries that produce bioethanol are also enriched in lignin [8]. Within these biorefineries, the residual lignins come either from the solid residues generated after saccharification/fermentation processes (lignin of lower quality) or from the liquid fractions generated in the pre-treatments carried out on the raw biomass (high purity lignin) [13]. If these lignins are also considered, an increase of up to 225 million tons/year of residual lignins is forecasted by 2030 [14].

Most of such lignin side streams are normally burned, due to their high calorific value, to obtain energy that is partially used in the same production plant. However, this process generates an excess of energy, making more interesting the valorization of the residual lignin into high value-added products that guarantee the profitability of these plants [15]. Interestingly, a wide range of products can be obtained from them with application in many different industries, given the interesting properties that these residual lignins may exhibit (e.g., biodegradability, antioxidant and antimicrobial capacity, high reinforcing ability, surfactant properties) and the possibility of chemical modifications due to the high amount of chemicals sites available in the lignin structure [16].

Among promising alternatives, lignin-derived products could find application in the asphalt industry that has demonstrated a strong commitment to the development of more sustainable technologies and materials. In this regard, reduced temperature asphalt technologies, involving cold or half warm mix asphalt, may lead to a remarkable reduction in energy consumption (up to 7.5 times lower) and emissions (up to 7 times less kg equivalent CO_2_/ton product) [17]. For such technologies, the use of a bituminous emulsion allows bitumen mixing with aggregate and further compaction operations to be performed at lower temperatures (in the range 60–130 °C) compared with those used for hot mix asphalt, which is typically above 165 °C [17,18,19]. Moreover, the development of oil-in-water bitumen emulsions stabilized by a biobased surfactant such as lignin would be clearly in line with the above commented sustainability approach.

However, markets and similar product specifications demand positively charged cationic bitumen emulsions [20], given that most common aggregates are of acidic type (i.e., siliceous-based derivatives) and exhibit a negative charge on their surfaces [21]. Due to the absence of protonatable functional groups, raw lignins cannot be used as cationic emulsifiers requiring their amination through the well-known Mannich reaction, which involves formaldehyde and amines as reagents, and this may be carried out under alkaline conditions [22,23]. The resultant modified lignins become positively charged and soluble under an acidic aqueous phase of the emulsion. These charges, when used in cationic bitumen emulsions for asphalt, are expected to stabilize the bitumen–water interface and, more interestingly, promote binder interaction and adhesion to a negatively charged surface of aggregates.

With this aim, this work compares the emulsification ability of three commercial lignins, by-products of the pulp and paper (kraft pulping) and bioethanol industries, which have been chemically modified via the Mannich reaction to be used as cationic emulsifiers. Initially, a comprehensive characterization of the different lignins has been carried out, and the emulsification ability of the resulting cationic surfactants has been assessed on prototype silicone oil-in-water emulsions in terms of droplet size distribution and viscous behavior. Afterwards, cationic bitumen emulsions stabilized by a modified lignin have been designed attending to the surfactant content and reagent molar ratio used for the amination reaction.

## 2. Materials and Methods

### 2.1. Raw Materials

A kraft lignin (referred to as KFT) from Sigma Aldrich (Madrid, Spain) and two lignin samples derived from the bioethanol process with two purification grades, provided by DONG Energy (Fredericia, Denmark) (referred to as BIOeth1 and BIOeth2, respectively), were used as precursors to obtain cationic lignins (C-KFT, C-BIOeth1 and C-BIOeth2). Bioethanol lignins are expected to be the solid residue fraction derived from wheat straw subjected to a hydrothermal pre-treatment followed by enzymatic hydrolysis and sugars fermentation to obtain bioethanol [24].

Lignin amination was performed following the Mannich reaction procedure, using tetraethylene pentamine (TEPA) and formaldehyde 37% as reagents, which were both supplied by Sigma Aldrich (Spain).

In the production of the model emulsions, silicone oil (FS100 from Esquim SA, Barcelona, Spain) with an approximate viscosity of 100 mPa·s at ambient temperature was chosen. This viscosity allowed us to easily reproduce at ambient conditions bitumen viscosity prior to its emulsification at high temperature, which is recommended to be less than 200 mPa·s before entering the colloid mill [20]. Subsequently, for the second part of the study, a bitumen with a penetration grade of 160–220 (Repsol SA, Madrid, Spain) was emulsified.

### 2.2. Preparations

Amination reaction was carried out under alkaline conditions at 60 °C for 4 h, previously dissolving lignin in distilled water at two pH values, 10 and 13. Formaldehyde (Fd) and tetraethylene pentamine (TEPA) were used as reagents for the amination of lignins (Lig) at three different Lig/TEPA/Fd stoichiometric molar ratios, 1/7/7, 1/14/14, and 1/28/28. Once reaction finished, the emulsification pH was reduced to 1 with hydrochloric acid (HCl) to activate new amine functional groups of modified lignins. Lignin amination by TEPA has been confirmed by FTIR analysis (as described below), suggesting that modification takes place with the inclusion of amine functional groups in the aromatic units [18].

The emulsification process of model silicone emulsions was performed with the IKA T25/25F homogenizer (Germany) at 20,000 rpm rotational speed for 4 min, and bitumen emulsification was carried out with an IKAT50/G45M at 10,000 rpm for 3 min. Silicone oil was emulsified at room temperature, whereas bitumen was blended with the aqueous phase (water and surfactant) to prepare a premixture at 90 °C prior to the emulsification stage. Two oily disperse phase (O) concentrations were studied, 60% and 70%, and cationic emulsifier (Surf) concentrations ranged from 0.25 to 0.75% Surf.

### 2.3. Tests and Measurements

Lignin composition was determined by standard analytical methods (National Renewable Energy Laboratory NREL/TP-510-42618). In order to determine the carbohydrate composition, samples were subjected to quantitative acidic hydrolysis in two steps. The obtained hydrolyzed liquids were then analyzed for sugar contents using an Agilent Technologies 1260 HPLC fitted to a refractive index detector and an Agilent Hi-PlexPb column operated at 70 °C with Milli-Q water as the mobile phase, which was pumped at a rate of 0.6 mL/min. Soluble lignin was also measured in the hydrolyzed liquids using a Lambda 365 UV/Vis spectrometer (Perkin Elmer, Boston, MA, USA) at 205 nm. The resulting solid residues obtained after the acid hydrolysis were considered acid-insoluble lignin (Klason lignin). Ash content was determined following the standard UNE 57050:2003. Finally, elemental analysis was conducted in an Elemental Thermo Flash EA1112 analyzer (Thermo Fisher Sci., MA, USA) following the standards EN 15104, EN 15296 and EN 15289. 

The total phenols content of lignins was analyzed according to Jiménez-López et al. [25]. For that, lignins were previously dissolved in dimethylsulfoxide (DMSO). Then, the absorbance of a mixture with Na_2_CO_3_, Folin–Ciocalteau reagent, and lignin solutions was measured at 760 nm using a UV–Vis spectrophotometer (Lambda 365, PerkinElmer, Boston, MA, USA). The total phenols content of samples was quantified using a calibration curve prepared from a standard solution of gallic acid (1–20 mg/L) and expressed as mg gallic acid equivalent (GAE)/g of lignin (on a dry basis).

Size exclusion chromatography analysis (SEC) was used to evaluate the average molecular weight (Mw), number average (Mn) and polydispersity (Mw/Mn) of the lignins. Lignin samples were examined by high-performance liquid chromatography (HPLC) using a column PLgel 10 µm MIXED B 300 × 7.5 mm operated at 70 °C. A column PLgel 10 µm guard 50 × 7.5 mm was also employed. N,N-dimethylformamide (DMF) pumped at a rate of 1 mL/min was employed as the mobile-phase. Polystyrene was used as the standard, with peak average molecular weights of 570, 8900, 62,500, 554,000 (Sigma-Aldrich, Spain). For detection, a G1362A refractive index (RI) detector (Agilent, Waldbronn, Germany) was used. Solubilization was almost complete; only approximately 5% of the samples remained undissolved, which it is considered a non-significant percentage of the samples for the determination of their molecular weight.

Fourier-transform infrared spectroscopy (FTIR) measurements were conducted for identifying the functional groups present in lignin samples. A JASCO FT/IR 460 Plus spectrometer (Jasco, Japan) was used to acquire the spectra from 4000 to 600 cm^−1^, after 400 scans at 1 cm^−1^ resolution.

Emulsion characterization was carried out by means of droplet size distribution measurements and viscosity curves at 30 °C. Particle size distribution tests were conducted in a Mastersizer 2000 Malvern (UK), at ambient temperature, according to the laser diffraction method. Steady flow viscous tests between 0.5 and 500 s^−1^ were performed using a profiled-surface coaxial cylinder (d_i_ = 26.657 mm and d_o_ = 28.922 mm), to avoid wall-slip phenomena [26], in a controlled stress rheometer Anton Paar MCR301 (Graz, Austria).

## 3. Results and Discussion

### 3.1. Lignin Characterization

Table 1 shows compositions obtained for the different raw lignin-rich products. As may be seen, the kraft lignin sample (KFT) shows a higher lignin concentration than the bioethanol-derived products (BIOeth1 and BIOeth2). This result is related to the higher concentrations of carbohydrate residues (around 23–24%) and ashes (9–10%) found for BIOeth1 and BIOeth2 samples. As known, the solid residual fraction after bioethanol production is enriched in lignin but with a certain contamination of carbohydrates derived from the non-hydrolyzed cellulose during the saccharification [8], among which glucose is the main sugar (Table 1).

During bioethanol production, hydrothermal pre-treatments solubilize a great part of hemicellulose fraction, resulting in pre-treated materials enriched mostly in cellulose and lignin [27]. This residual lignin in the pre-treated materials may unspecifically bind hydrolytic enzymes during the subsequent enzymatic hydrolysis [28], decreasing the saccharification yields and, consequently, increasing the content of carbohydrates non-hydrolyzed (mainly glucose) in bioethanol-derived lignin samples. Regarding the kraft lignin sample, the small amount of carbohydrates found (mainly xylose) is normally attributed to the lignin carbohydrates complexes and non-bounded sugars, as suggested by Alekhina et al. [29] and dos Santos et al. [30]. These authors have also described that the fast degradation of carbohydrates (mainly hemicelluloses as xylan) during kraft pulping, through the well-known peeling reactions, together with the low solubility of hemicelluloses in acid media during lignin precipitation from black liquors, are the factors that explain why xylose is the most abundant sugar in KFT (Table 1).

As for the higher ash content found for bioethanol-derived lignin samples compared with KFT (Table 1), the presence of ashes in kraft lignin is probably related to the inorganic compounds used in kraft pulping (e.g., NaOH and Na_2_S) and the high amount of Na_2_SO_4_ salts formed during the acid precipitation step. However, the low content of ashes found for this lignin would indicate it has been subjected to a purification post-treatment. Conversely, the higher ash concentration observed for BIOeth1 and BIOeth2 samples cannot be explained by the isolation process of these lignins, where neither kraft cooking nor acid precipitation processes have been used. The reason for the higher ash concentration found in bioethanol lignins could be related to the content of inorganic compounds present in the raw material (e.g., wheat straw). It is known that the ash and silicate contents of different non-woody materials, such as wheat straw, are higher than in wood lignin [31]. In any case, the ash content would depend on post-treatments performed on the lignin-rich product, such as salt removal with a wash step using deionized water until pH 5 [29].

Another relevant difference observed among samples was the lower sulfur content measured in bioethanol samples, which is 10 times lower than that found in KFT (Table 1). This result is due to the use of Na_2_S during the kraft process, in which lignocellulosic raw material is treated with NaOH and Na_2_S in an aqueous solution at high temperature [32,33]. As a result, some ionizable groups as phenolic hydroxyls present in kraft lignin may become sulfonated during kraft pulping.

Regarding acid soluble lignin contents (Table 1), BIOeth1 and BIOeth2 lignin samples presented a lower amount than kraft lignin. This fact could be attributed to the hydrothermal pre-treatment used in bioethanol production that could have removed low molecular weight degradation products and hydrophilic derivatives of lignin, which are both components of the acid-soluble lignin [27]. On the other hand, the severity used during the kraft pulping along with the lignin precipitation step at low pH would favor the increase in the acid-soluble lignin content found in KFT [34].

**Figure 1:**
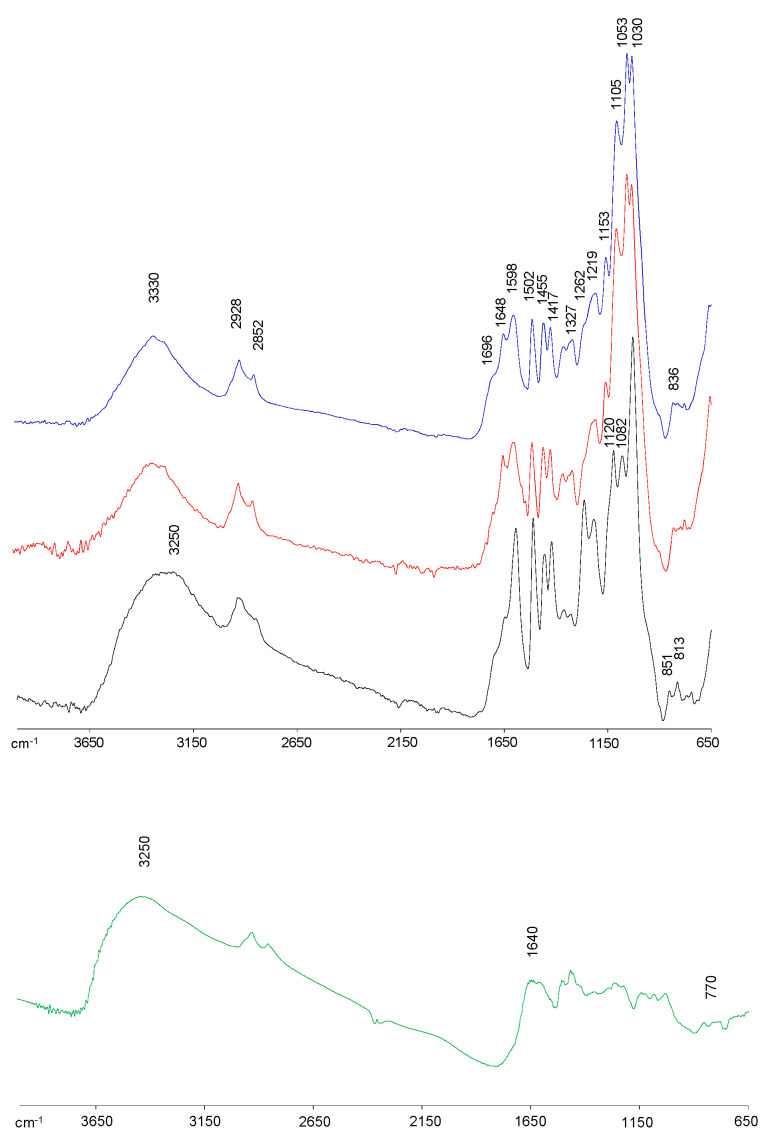
FTIR spectra, 4000–650 cm^−1^ region, of KFT (black), BIOeth1 (red) and BIOeth2 (blue) lignin samples, and KFT lignin sample after amination reaction (green).

FTIR spectroscopy was used to characterize the main functional components of lignin samples (Figure 1), showing all spectra the typical lignin bands according to previous studies [8,9,29,35]. At 3330 cm^−1^, a wide band attributed to O−H stretching vibration (both alcohol and phenolic hydroxyl groups) was displayed in all lignin spectra. The kraft lignin spectrum showed a broader band, moved toward 3250 cm^−1^, with higher intensity compared with bioethanol-derived lignin spectra, demonstrating a higher content of hydroxyl groups, mainly phenolic, possibly because of the major cleavage of alkyl–aryl ether linkages (β-O-4′ substructures) during the kraft pulping process [29,36]. Thus, the phenol content measured on samples agrees with this result, having KFT, BIOeth1 and BIOeth2, respectively, 366, 102 and 104 mg GAE/lignin. The bands at 2928 cm^−1^ and 2852 cm^−1^ correspond to the C−H stretching vibration in the −CH_3_ and −CH_2_− groups, respectively, whereas the band at 1455 cm^−1^ is associated to the C−H asymmetric vibrations and deformation. The intensity of these bands was lower for kraft lignin compared with bioethanol-derived lignins, which could be related to a lower aliphatic group content, which is probably due to a higher aliphatic chain shortening during kraft pulping [36]. The higher content of hydroxyl groups, together with the aliphatic chain shortening observed in the kraft lignin spectrum, suggests a lignin with a major fragmented structure compared with bioethanol-derived lignins. Similar effects were described by Santos et al. [8] when bioethanol lignins from olive tree pruning were compared with alkaline lignins, either soda-anthraquinone or kraft lignins, the latter being more degraded.

All lignin spectra presented the typical bands at 1598, 1502, and 1417 cm^−1^ attributed to vibrations of lignin aromatic skeleton. Furthermore, the three spectra showed a shoulder at 1648 cm^−1^ (conjugated C=O groups), especially visible in bioethanol lignins, which could be explained by lignin oxidation. Nevertheless, in the case of bioethanol lignins, these carbonyl groups may also be associated to the C=O stretching of amide bonds from cellulolytic enzymes and/or biological contaminations from the fermentative microorganisms [37,38,39]. This last hypothesis is supported by the higher nitrogen content found in bioethanol lignins samples (Table 1). Another shoulder at 1698 cm^−1^, associated to unconjugated C=O groups stretching from lignin oxidation, was also observed in all spectra, although it can also be attributed to carbonyl groups in hemicelluloses impurities [29].

Regarding the presence of S, G or H units, the kraft lignin spectrum showed the typical pattern of softwood lignins, with pronounced bands at 1262 cm^−1^ (G ring breathing with C=O stretching), 1219 cm^−1^ (G ring breathing with C–C, C–O, and C=O stretching), 1030 cm^−1^ (C–H bond deformation in G units) and the bands at 853 cm^−1^ and 817 cm^−1^ (C–H out of plane in position 2, 5 and 6 of G units) [29]. On the other hand, bioethanol-derived lignins showed the corresponding G units bands, together with S and H units bands at 1327 cm^−1^ (S aromatic ring breathing) and at 836 cm^−1^ (C–H out of plane in position 2 and 6 of S units and all positions of H units), which are typical of hardwood lignins as well as non-woody lignins (e.g., wheat straw) [8,9,35].

In agreement with the higher carbohydrate content (mainly glucose) determined in bioethanol lignins (Table 1), their spectra displayed cellulose bands at 1153, 1105, 1053, and 1030 cm^−1^ overlapping some lignin bands. On the other hand, the kraft lignin spectrum showed a new band at 1082 cm^−1^, which is associated to hemicelluloses. Furthermore, as previously commented, the band intensity at 1698 cm^−1^ could also be partly correlated to hemicelluloses.

After amination reaction, the structural units appear different as observed by the following evidence on the modified Kraft lignin spectrum: (a) a broader and stronger band at 3250 cm^−1^, in which N-H stretching vibrations could be contributing, as well as the presence of a newly developed peak centered at 770 cm^−1^ associated to N-H wagging out of plane; (b) a broader band appeared at 1640 cm^−1^, arising from the N-H bending vibrations in a single bond NH_2_ structure; and (c) the C–H vibrations from the aromatic skeleton of lignin showed a lesser definition, such as 1598, 1502, 1455 and 1417 cm^−1^ [18,40,41]. This would suggest that modification, with the inclusion of an amine functional group, has occurred in their aromatic unit.

**Figure 2:**
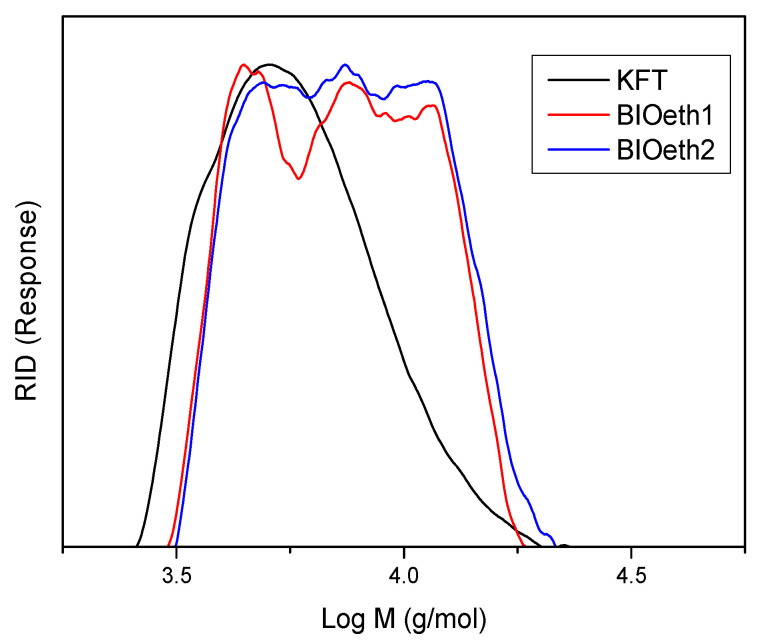
Molecular weight distribution of KFT (black), BIOeth1 (red) and BIOeth2 (blue) lignin samples.

Finally, Figure 2 and Table 2 show, respectively, lignin molecular weight distributions and their average molecular weight (Mw), number average (Mn), and polydispersity (Mw/Mn). As seen in Table 2, all lignins showed low molecular weights with very similar polydispersity values. Nevertheless, the kraft lignin sample showed a lower molecular weight than lignin samples from bioethanol production (BIOeth1 and BIOeth2). The lower molecular weight of kraft lignin corroborates with the higher phenolic content observed by the FTIR spectroscopy of this lignin, compared with bioethanol lignins, which supports a major lignin depolymerization during the kraft pulping process. In this sense, previous studies indicated that the molecular weight of kraft lignin could be greatly reduced due to an extensive cleavage of alkyl-aryl ether linkages of β-O-4′ bonds during kraft pulping [29,42]. On the contrary, lignins from bioethanol production are usually found to be less degraded [8].

### 3.2. Lignin as Cationic Surfactants of Model Emulsions

**Figure 3:**
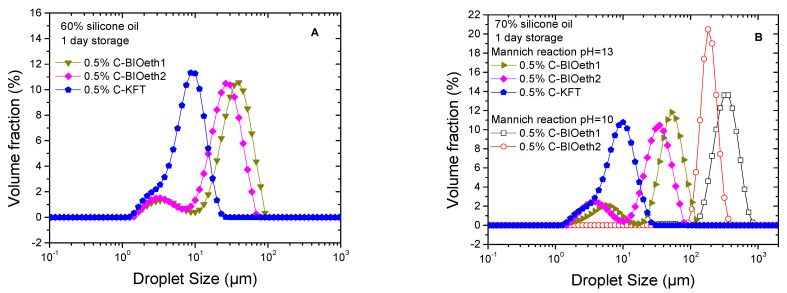
Droplet size distribution of emulsions formulated with 60% (**A**) and 70% silicone oil (**B**) and stabilized by 0.5% surfactant. Emulsification ability of cationic surfactants as a function of their lignin source and pH of amination.

Emulsion properties such as droplet size distribution (DSD) and rheology are closely linked to emulsifier performance. Optimization of the emulsifier was initially carried out on model silicone–oil-based emulsions by studying the effect of the lignin source and reaction pH for a selected reagent ratio of 1/14/14 (Lig/TEPA/Fd reagents), which was calculated according to the previous lignin characterization. Figure 3 shows the effect of lignin source on the emulsification ability of the cationic surfactants prepared under alkaline conditions (pH = 10 and 13). When reaction at pH of 13 was used for lignin amination, no matter the selected lignin source, a 0.5% cationic lignin was able to stabilize the 60% and 70% oil emulsions formulated at pH = 1. Systems emulsified by bioethanol-derived cationic surfactants (C-BIOeth1 and C-BIOeth2) showed bimodal distributions, obtaining a narrower droplet size distribution (DSD) for the emulsions stabilized by C-BIOeth2 (i.e., the most purified lignin by-product from the bioethanol process). Conversely, emulsion stabilized by cationic kraft lignin (C-KFT) showed a monomodal DSD with a shoulder at low particle size.

The mean particle size of emulsions was determined as Sauter (*D*_3,2_) and De-Brouckere or volumetric (*D*_4,3_) diameters [43]:

(1) $$D_{3,2}=\frac{\sum_{i}n_{i}d_{i}^{3}}{\sum_{i}n_{i}d_{i}^{2}},$$

(2) $$D_{4,3}=\frac{\sum_{i}n_{i}d_{i}^{4}}{\sum_{i}n_{i}d_{i}^{3}}$$

where *n_i_* is the number of droplets with diameter *d_i_*. Table 3 shows that C-KFT leads to emulsions with much lower Sauter and volumetric mean diameters. Furthermore, an increase in oil concentration from 60% to 70% hardly modifies the values of *D*_3,2_ and *D*_4,3_ no matter the lignin considered. Likewise, the emulsion particle size remained almost constant after one week of storage time at room temperature (Table 3). Conversely, a less alkaline medium (pH = 10) for the amination reaction significantly reduced lignin’s emulsification ability, shifting DSD curves toward higher droplet sizes (Figure 3B and Table 3).

**Figure 4:**
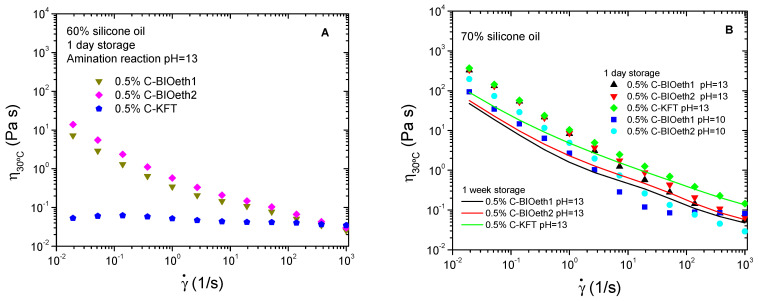
Viscous behavior of silicone emulsions stabilized by cationic lignin. Effect of the lignin source of cationic surfactants synthetized, pH of amination reaction and storage time on emulsions containing 60% (**A**) and 70% oil (**B**).

The viscous behavior of prepared emulsions is shown in Figure 4. Systems presented a shear thinning non-Newtonian behavior characterized by a power-law decrease in viscosity with shear rate, which is more evident for the most oil-concentrated (and, therefore, more viscous) emulsions (Figure 4B). This behavior is well known in flocculated emulsions, in which the slope of the shear-thinning region is higher as the flocculation degree increases (Santos et al., 2015). Thus, Figure 4B shows, for cationic lignins synthetized at pH13, that emulsions stabilized by bioethanol-derived lignins present viscosity curves steeper (i.e., more dependent on shear rate) than C-KFT-stabilized systems. However, a decrease in the slopes of the shear-thinning region is found with storage time, suggesting a weakening of flocculation degree and a slow destabilization process of the emulsion that, instead, does not lead to an eventual droplet coalescence, as may be deduced from Table 3.

Interestingly, when the oil concentration is 60%, which is far below the expected maximum packing fraction for the emulsion disperse phase, cationic kraft lignin leads to weakly flocculated emulsions as may be deduced from the almost Newtonian behavior exhibited by this system, unlike bioethanol-derived cationic lignins (Figure 4A). Flocculation is known to increase the apparent dispersed phase volume, along with the formation of non-spherical aggregates. Both factors may contribute to the development of a non-Newtonian viscous response [44]. Under such conditions, with a less packed disperse phase in the emulsion (i.e., with a higher distance among droplets), only C-BIOeth1 and C-BIOeth2 would be able to build up an extended three-dimensional network formed by droplets interconnected by the lignin surfactant. This network seems to be more developed for the C-BIOeth2 stabilized emulsion as may be deduced from its steeper viscous flow curve (Figure 3A).

Results obtained would be in good agreement with the structures and compositions of the above-described lignin-rich products. Thus, cationic surfactants obtained from bioethanol process are expected to have a higher molecular weight (Table 2) than kraft lignin cationic-derived surfactant. Likewise, the contamination of these lignins by sugars may have a strong influence on the emulsion rheology, since polysaccharides are known to have a thickening effect with little surface activity, increasing the viscosity of the continuous aqueous phase of the emulsions [44,45]. Together, the higher lignin molecular weight and the thickening effect of carbohydrate contamination are expected to favor droplet flocculation.

Conversely, the lower molecular weight of kraft lignin (with a more fragmented structure compared with bioethanol-derived lignins) would lead to less flocculated systems with a lower droplet size, suggesting a higher interfacial activity for C-KFT. This fact, together with its higher purification degree, with about 94% lignin concentration (Table 1), makes cationic kraft lignin the best candidate to stabilize bitumen emulsions.

### 3.3. Formulation of Lignin-Based Cationic Bituminous Emulsions

**Figure 5:**
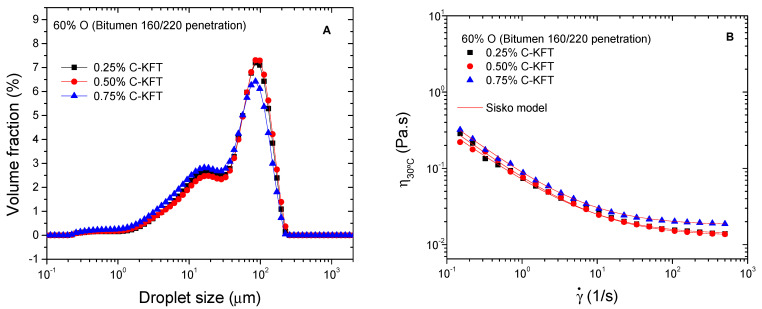
Effect of C-KFT concentration on droplet size distribution (**A**) and viscosity (**B**) of 60% bitumen emulsions.

According to the previous results, cationic kraft lignin showed the best performance as an emulsifier of silicone oil model emulsions, being selected as a potential cationic surfactant for bitumen emulsions. Regarding its surfactant activity, previously, it was found that the surface tension of this cationic lignin decreases with the increase in concentration up to 0.625% surfactant, and both polar and dispersive components of surface energy remain constant for higher concentrations [19]. As a result, the effect of surfactant concentration was initially assessed for the design of such emulsions in the range of 0.25–0.75%. Figure 5A shows that C-KFT successfully stabilizes water-in-oil emulsion containing 60% bitumen (O) at surfactant concentrations between 0.25 and 0.75%. In all cases, DSD curves show a trend to a bimodal distribution with peaks located at about 15 and 95 µm. Likewise, even though mean droplet diameters are similar for the three surfactant concentrations (Table 4), the smallest values of *D*_3,2_ and *D*_4,3_ are found for the emulsion stabilized with 0.75% C-KFT. All emulsions remained visually stable for at least one week, which is the shortest storage time required for this type of emulsions to be used in road applications.

Similarly, emulsion viscosities hardly change with surfactant concentration (Figure 5B). All flow curves display a non-Newtonian behavior with a shear thinning region at low shear rates followed by a trend to reach a high-shear-rate limiting viscosity, which corresponds to the shear-induced deflocculation process. The observed viscous behavior may be described by the Sisko model:

(3) $$\eta =\eta_{\infty }+k{\dot{\gamma }}^{n-1},$$

where *k* and *n* are, respectively, the consistency and flow indexes, and *η*_∞_ is the high-shear-rate limiting viscosity. As may be seen in Table 4, the Sisko model parameters slightly change with surfactant concentration, showing a trend to decrease flow index values with increasing concentration. Compared with previous model emulsions containing the same oil concentration, bituminous emulsions present higher viscosity and a more developed non-Newtonian character, which is characteristic of a more complex microstructure.

**Figure 6:**
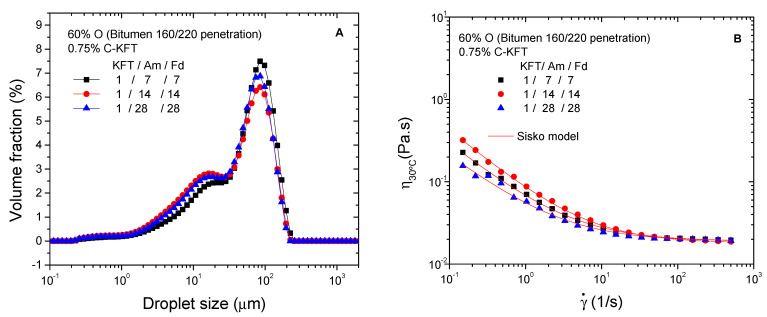
Effect of reagent molar ratio on droplet size distribution (**A**) and viscosity (**B**) of emulsions containing 60% bitumen and 0.75% C-KFT.

Finally, with the aim of optimizing surfactant properties, the effect of the reagent molar ratios selected for lignin aminations was assessed with two additional ratios, below and above the previously studied reagent ratio 1/14/14 (MKL/Am/Fd). As may be seen in Figure 6A, an increase in reagent ratios from 1/7/7 to 1/28/28 does not lead to remarkable changes in DSD curves, although the number of small droplets tends to increase above 1/7/7, with the development of the peak located around 15 µm. Interestingly, the smallest mean droplet diameters were obtained for a reagent ratio of 1/14/14 (Table 5).

A shear-thinning behavior followed by a constant high shear-limiting viscosity was found for all samples (Figure 6B). Comparing the values of Sisko model parameters (Table 5), high-shear-rate limiting viscosities are hardly affected by reagent stoichiometric. The highest consistency index (k) and slope for the shear-thinning region (i.e., the lowest value of n) were found for the surfactant synthetized with a molar ratio of 1/14/14, whilst the lowest ones corresponded to the system stabilized with a surfactant prepared with a 1/28/28 reagent ratio. In any case, all reactions, with stoichiometric values between 1/7/7 and 1/28/28, were considered successful as deduced from emulsifier solubility under the acidic environment and from the already demonstrated performance as emulsifiers.

## 4. Conclusions

The kraft lignin used in this work as a source of cationic surfactants is characterized by a high purity: about 94% lignin concentration. Conversely, less purified bioethanol-derived samples, with a lignin content around 65%, also contain high concentrations of carbohydrate residues (23–24%) and ashes (9–10%). FTIR spectroscopy conducted on kraft lignin, compared with bioethanol-derived products, supports a major lignin depolymerization during the kraft pulping process, showing a lower content of aliphatic groups and higher content of hydroxyl groups. As a result, kraft lignin presents a more fragmented structure with a lower molecular weight.

On these grounds, the cationic surfactants obtained from the bioethanol process are expected to have a higher molecular weight than the kraft lignin cationic surfactant, which, together with the thickening effect of carbohydrate contamination, would favor droplet flocculation. The resultant emulsions would be stabilized by a combination of surfactant interfacial (electrostatic) interactions, due to the positive charge of the protonated amine groups, and steric interactions related to the high molecular weight of lignin and the presence of carbohydrates located in the emulsion continuous phase. A decrease in the alkalinity used for the amination reaction of these lignins, from pH = 13 to 10, did not improve their performance as cationic emulsifiers, leading to dispersions with large droplet sizes.

Conversely, the lower molecular weight and higher purification degree of raw kraft lignin has led to less flocculated emulsions with a lower droplet size, suggesting a higher interfacial activity for C-KFT. As a result, cationic kraft lignin has been selected as the best candidate to stabilize bitumen emulsions.

C-KFT has successfully stabilized oil-in-water emulsions containing 60% bitumen, with surfactant concentrations between 0.25 and 0.75%. In all cases, DSD curves have shown a trend to a bimodal distribution with peaks located at about 15 and 95 µm, and emulsions exhibited a non-Newtonian flow behavior, with a shear-thinning region followed by a trend to reach a constant viscosity at a high shear rate. Amination reactions, performed at pH = 13 and stoichiometries between 1/7/7 and 1/28/28, were considered successful as deduced from the solubility of the modified (cationic) emulsifier under an acidic environment (aqueous phase of the emulsion) and their demonstrated performance as cationic emulsifiers. In this sense, a more depolymerized lignin (i.e., with lower molecular weight) would result in a surfactant that has a higher positive charge per gram added to the emulsion. That weight of emulsifier would be composed by more molecules of lignin with protonated amine groups.

## Figures and Tables

**Table 1:** Composition of the different raw lignin-rich products.

Sample	Klason Lignin (wt.%)	Soluble Lignin (wt.%)	Total Lignin (wt.%)	Glucose (wt.%)	Xylose (wt.%)	Arabinose (wt.%)	Ash (wt.%)	Elemental Analysis (wt.%)				
								C	H	N	O	S
KFT	89.08	5.14	94.22	0.92	1.61	0.20	2.4	61.2	6.72	2.18	26.9	1.8
BIOeth1	60.59	3.35	63.94	23.61	5.68	0.26	10.4	49.1	5.16	2.37	33.4	0.13
BIOeth2	62.12	3.62	65.74	24.06	5.49	0.17	9.4	49.0	5.21	2.75	34.0	0.12

**Table 2:** Average molecular weight (Mw), number average (Mn) and polydispersity (Mw/Mn) of the lignin samples.

Sample	Mn (g/mol)	Mw (g/mol)	Polydispersity
KFT	5366	6248	1.164
BIOeth1	6618	7869	1.189
BIOeth2	6878	8217	1.195

**Table 3:** Mean droplet diameter of cationic silicone emulsions prepared at pH = 1 and with a 0.5% surfactant (reagent molar ratio 1/14/14).

Lignin Type	Reaction pH	Oil Conc. (wt.%)	*D*_3,2_ (µm)		*D*_4,3_ (µm)	
			Storage Time		Storage Time	
			1 Day	1 Week	1 Day	1 Week
BIOeth1	13	60	16.9	-	37.3	-
BIOeth2	13	60	14.2	-	27.4	-
KFT	13	60	6.8	-	9.2	-
BIOeth1	10	70	311.9	-	376.5	-
BIOeth2	10	70	194.3	-	207.5	-
BIOeth1	13	70	18.7	17.3	49.7	49.3
BIOeth2	13	70	12.8	12.9	31.0	32.9
KFT	13	70	7.3	7.8	10.0	10.5

**Table 4:** Mean droplet diameter and Sisko model parameters of 60% bitumen emulsions as a function of C-KFT concentration (reagent molar ratio 1/14/14).

Surfactant Conc. (wt.%)	*D*_3,2_ (µm)	*D*_4,3_ (µm)	*η*_∞_ (Pa s)	*k* (Pa s^n^)	*n* (-)
0.25	11.7	60.9	0.014	0.06	0.29
0.50	11.4	63.7	0.013	0.06	0.27
0.75	9.1	53.9	0.018	0.07	0.23

**Table 5:** Mean droplet diameters and Sisko model parameter of 60% bitumen emulsions as a function of C-KFT reagent molar ratio.

Reagent Molar Ratio (KFT/TEPA/Fd)	*D*_3,2_ (µm)	*D*_4,3_ (µm)	*η*_∞_ (Pa s)	*k* (Pa s^n^)	*n* (-)
1/7/7	12.20	63.84	0.019	0.05	0.25
1/14/14	9.12	53.85	0.018	0.07	0.23
1/28/28	10.24	55.10	0.019	0.04	0.28

## Data Availability

The data presented in this study are available on request from the corresponding author.
